# Assessing duplication and loss of *APETALA1/FRUITFULL* homologs in Ranunculales

**DOI:** 10.3389/fpls.2013.00358

**Published:** 2013-09-17

**Authors:** Natalia Pabón-Mora, Oriane Hidalgo, Stefan Gleissberg, Amy Litt

**Affiliations:** ^1^Grupo de Biotecnología, Instituto de Biología, Universidad de AntioquiaMedellín, Colombia; ^2^The New York Botanical GardenBronx, NY, USA; ^3^Laboratori de Botànica, Facultat de Farmàcia, Universitat de BarcelonaBarcelona, Spain; ^4^38 Fairview AvenueAthens, OH, USA

**Keywords:** gene duplication, *APETALA1*, *FRUITFULL*, basal-eudicots, *FRUITFULL-like*, Ranunculales

## Abstract

Gene duplication and loss provide raw material for evolutionary change within organismal lineages as functional diversification of gene copies provide a mechanism for phenotypic variation. Here we focus on the *APETALA1*/*FRUITFULL* MADS-box gene lineage evolution. *AP1/FUL* genes are angiosperm-specific and have undergone several duplications. By far the most significant one is the core-eudicot duplication resulting in the *euAP1* and *euFUL* clades. Functional characterization of several *euAP1* and *euFUL* genes has shown that both function in proper floral meristem identity, and axillary meristem repression. Independently, *euAP1* genes function in floral meristem and sepal identity, whereas *euFUL* genes control phase transition, cauline leaf growth, compound leaf morphogenesis and fruit development. Significant functional variation has been detected in the function of pre-duplication basal-eudicot *FUL-like* genes, but the underlying mechanisms for change have not been identified. *FUL-like* genes in the Papaveraceae encode all functions reported for *euAP1* and *euFUL* genes, whereas *FUL-like* genes in *Aquilegia* (Ranunculaceae) function in inflorescence development and leaf complexity, but not in flower or fruit development. Here we isolated *FUL-like* genes across the Ranunculales and used phylogenetic approaches to analyze their evolutionary history. We identified an early duplication resulting in the *RanFL1* and *RanFL2* clades. *RanFL1* genes were present in all the families sampled and are mostly under strong negative selection in the MADS, I and K domains. *RanFL2* genes were only identified from Eupteleaceae, Papaveraceae s.l., Menispermaceae and Ranunculaceae and show relaxed purifying selection at the I and K domains. We discuss how asymmetric sequence diversification, new motifs, differences in codon substitutions and likely protein-protein interactions resulting from this Ranunculiid-specific duplication can help explain the functional differences among basal-eudicot *FUL-like* genes.

## Introduction

**Figure 1:**
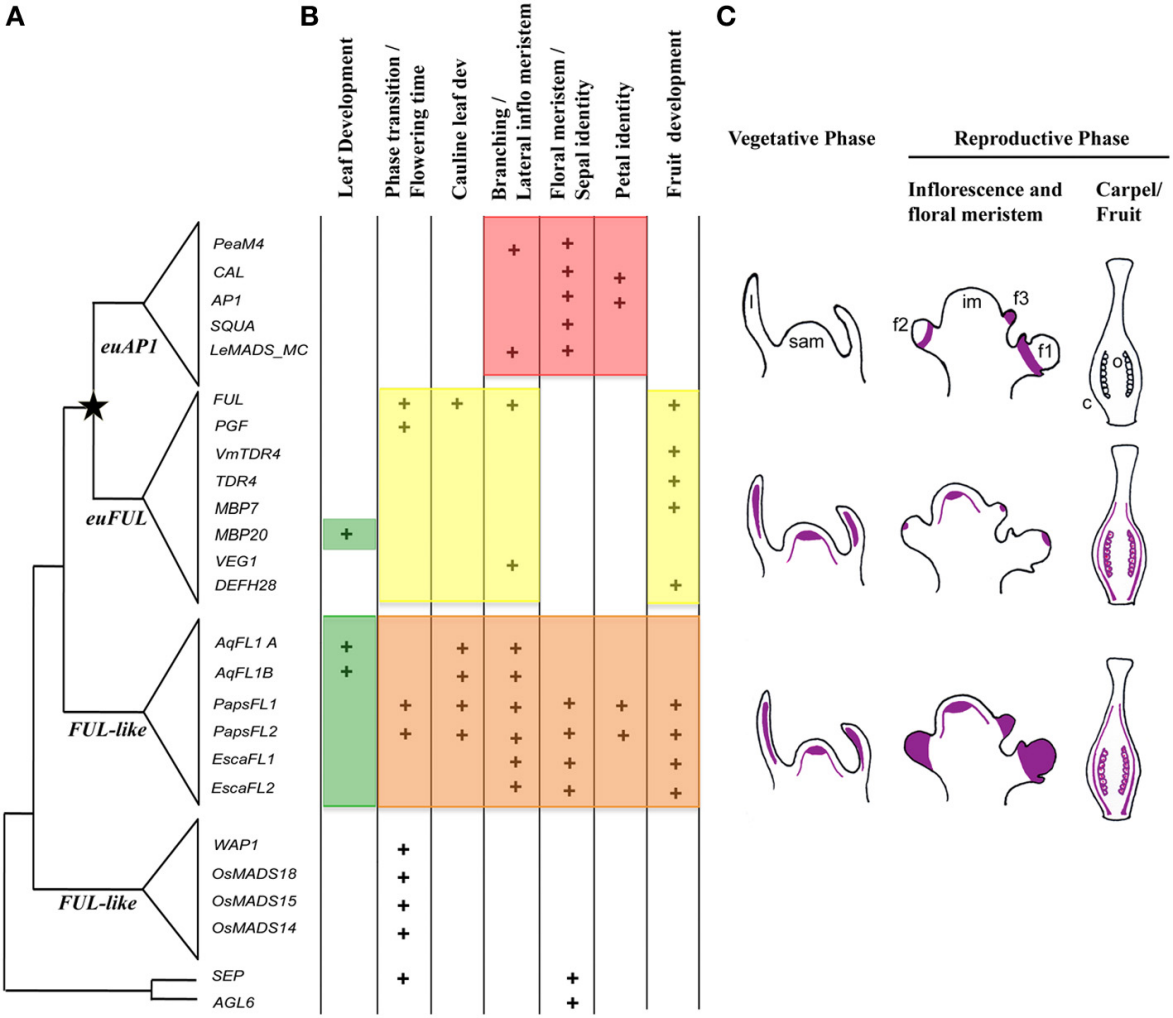
**Summary of: (A) duplication events, (B) functional evolution and (C) expression patterns of *APETALA1/FRUITFULL* homologs in angiosperms**. **(A)** Gene tree showing a major duplication (star) coinciding with the diversification of core-eudicots resulting in the *euAP1* and the *euFUL* clades. The pre-duplication genes in basal eudicots, monocots and basal angiosperms are more similar in sequence to the *euFUL* genes and thus have been named the *FUL-like* genes. To the right of the tree are the genes that have been functionally characterized. In core-eudicots: *PeaM4* and *VEG1* from *Pisum sativum* (Berbel et al., 2001, 2012), *CAL, AP1* and *FUL* from *Arabidopsis thaliana* (Ferrándiz et al., 2000), *SQUA* and *DEFH28* from *Antirrhinum majus* (Müller et al., 2001), *LeMADS_MC, TDR4, MBP7, MBP20* from *Solanum lycopersicum* (Vrebalov et al., 2002; Bemer et al., 2012; Burko et al., 2013), *PGF* from *Petunia hybrida* (Immink et al., 1999), and *VmTDR4* from *Vaccinium myrtillus* (Jaakola et al., 2010). *AGL79* is the *Arabidopsis FUL* paralog within the *euFUL* clade, however, it was not included in the figure because it has not been functionally characterized yet. In basal eudicots: *AqFL1A* and *B* from *Aquilegia, PapsFL1* and *FL2* from *Papaver somniferum* and *EscaFL1* and *FL2* from *Eschscholzia californica* (Pabón-Mora et al., 2012, 2013). In monocots: *WAP1* in *Triticum aestivum* (Murai et al., 2003), *OsMADS18, 14, 15* in *Oryza sativa* (Moon et al., 1999; Kobayashi et al., 2012). **(B)** Summary of the functions reported for *AP1/FUL* homologs. Each plus-sign means that the function has been reported for a particular gene. The orange color highlights the pleiotropic roles of ranunculid *FUL-like* genes ancestral to the core-eudicot duplication. Red and yellow highlight the separate functions that core-eudicot homologs have taken on. Green indicates the newly identified role of *FUL-like* genes in leaf morphogenesis in *Aquilegia* and in *Solanum*. **(C)** Summary of gene expression patterns of *AP1/FUL* homologs during the vegetative and reproductive phases. The purple color indicates the areas where expression for each gene clade has been consistently reported (Immink et al., 1999; Moon et al., 1999; Ferrándiz et al., 2000; Müller et al., 2001; Berbel et al., 2001, 2012; Vrebalov et al., 2002; Murai et al., 2003; Jaakola et al., 2010; Bemer et al., 2012; Pabón-Mora et al., 2012, 2013; Burko et al., 2013). c, carpel; f1, flower plastochron 1 with sepal and petal primordia; f2, old floral meristem 2; f3, young floral meristem 3; im, inflorescence meristem; l, leaf; sam, shoot apical meristem; o, ovules.

The *APETALA1/FRUITFULL* genes are best known for the roles of *APETALA1* (*AP1*), *CAULIFLOWER* (*CAL*) and *FRUITFULL* (*FUL*) paralogs in *Arabidopsis thaliana*. Altogether *AP1, CAL* and *FUL* are responsible for proper floral meristem identity (Ferrándiz et al., 2000); in addition, *AP1* plays a key role promoting perianth identity. Because of this, it was included as an A-function gene in the ABC model of flower development (Irish and Sussex, 1990; Coen and Meyerowitz, 1991; Bowman et al., 1993; Gustafson-Brown et al., 1994; Ferrándiz et al., 2000). *CAL* is mostly redundant with *AP1*, however, it has been shown to play an independent role in petal formation (Kempin et al., 1995; Castillejo et al., 2005). *FUL* plays unique roles in proper cauline leaf development and fruit development, and is also a key factor in meristem maintenance and branching (Mandel and Yanofsky, 1995; Gu et al., 1998; Melzer et al., 2008). A fourth, less studied paralog, *AGL79*, is highly divergent in sequence and only expressed in roots, and it has not been functionally characterized (Parenicová et al., 2003). These paralogous genes are the result of duplications in the *AP1/FUL* gene lineage: whereas the origin of *AP1* and *FUL* is the result of a duplication that resulted in the *euAP1* and *euFUL* gene clades coincident with the origin of the core-eudicots, the close paralogs *AP1* and *CAL* are likely the result of genome duplication events correlated with the diversification of the Brassicaceae (Blanc et al., 2003; Bowers et al., 2003; Alvarez-Buylla et al., 2006; Barker et al., 2009; Figure 1A).

The core-eudicot duplication was followed by sequence changes in euAP1 proteins that produced a transcription activation (Cho et al., 1999) and a protein modification motif (Yalovsky et al., 2000). euFUL proteins instead retained the six hydrophobic amino-acid motif that is characteristic of pre-duplication proteins (FUL-like proteins). The function of this motif is unknown (Litt and Irish, 2003; Figure 1A). Together *euAP1* and *euFUL* genes promote floral meristem identity (Huijser et al., 1992; Berbel et al., 2001; Vrebalov et al., 2002; Benlloch et al., 2006). Additionally, *euAP1* genes play a unique role in the specification of sepal (and in *Arabidopsis*, petal) identity (Berbel et al., 2001; Vrebalov et al., 2002; Benlloch et al., 2006) whereas, *euFUL* genes function in the reproductive phase transition, proper cauline leaf development, branching, and fruit development as well as compound leaf development (Immink et al., 1999; Müller et al., 2001; Jaakola et al., 2010; Bemer et al., 2012; Berbel et al., 2012; Torti et al., 2012; Burko et al., 2013; Meyer et al., unpublished data; Figure 1B). The functional differences between *euAP1* and *euFUL* genes suggest an evolutionary scenario of either sub- or neofunctionalization after duplication, and studies of the function of *FUL*-like genes in basal eudicot Ranunculales (ranunculids) evaluated these two hypotheses. The *FUL-like* genes of *Papaver somniferum* (opium poppy; Papaveraceae) were shown to play pleiotropic roles that include essentially all those reported for *euAP1* and *euFUL* genes; thus, sub-functionalization was postulated as the outcome of the core-eudicot *AP1/FUL* duplication (Figure 1B; Pabón-Mora et al., 2012). However, functional analyses in *E. californica* (California poppy), also in Papaveraceae, showed that *FUL-like* genes in this species are involved only in a subset of these functions (Figure 1B: Pabón-Mora et al., 2012), and studies of *FUL-like* gene function in *Aquilegia coerulea* (columbine; Ranunculaceae) have shown only a role in regulating inflorescence branching and a role in compound leaf morphogenesis (Pabón-Mora et al., 2013; Figure 1B).

These studies on the *FUL-like* genes of ranunculids detected significant variation in the function of basal eudicot *FUL-like* genes. This observed functional diversity is not associated with changes in expression; in general all the ranunculid *FUL-like* genes are turned on in the shoot apical meristem and leaves, and expression is maintained throughout inflorescence and flower development, in all floral organs and fruit (Figure 1C). Thus, functional differences may instead be the result of protein sequence changes leading to differences in interactions with other transcription factors or downstream factors. Such sequence changes may hold clues to observed differences in function among genes belonging to different taxa (e.g., Papaveraceae vs. Ranunculaceae) as well as to the selective forces operating on genes of different paralagous lineages. Changes in developmental functions among paralogous genes are often accompanied by changes in rates and patterns of sequence evolution among loci (Purugganan and Suddith, 1998; Lawton-Rauh et al., 1999) and faster rates of evolution are usually associated with the occurrence of genetic redundancy (Lawton-Rauh et al., 1999). To understand functional evolution within ranunculid *FUL-like* genes this study had two main goals: (1) to explore in detail *FUL-like* gene duplications and losses in Ranunculales to establish the relationship among functionally characterized copies, and (2) to investigate differences in protein motifs and rates of evolution and selection across *FUL-like* genes in members of the ranunculids. The results of these analyses were used to understand the variation in *FUL-like* gene function among poppy, California poppy, and columbine and to identify changes in protein evolution that may be linked with differences in protein interaction capabilities across ranunculid FUL-like proteins.

## Materials and methods

### Plant material

Leaf and floral tissue was obtained from a number of basal eudicots, mostly within Papaveraceae s.l., Berberidaceae and Ranunculaceae, as well as non-eudicots within Aristolochiaceae (Piperales). Fresh material was obtained from living collections at The New York Botanical Garden, Bronx, NY or at the Systematics Garden at Lehman College, Bronx, NY. Voucher information for all species is listed in Table S1.

### Cloning and characterization of *FUL-like* genes

Total RNA was extracted from 0.5–1 g of young leaf or floral buds using TRIZOL reagent (Invitrogen) and was DNaseI-treated (Roche) to remove residual genomic DNA. 2 μg were used as template for cDNA synthesis with SuperScript III reverse transcriptase (Invitrogen) according to the manufacturer's instructions using the OligodT primer supplied. The resulting cDNA was diluted 1:10 for use in amplification reactions. Initial amplifications using degenerate primers to recover a pool of MADS-box genes were done as in Litt and Irish (2003), with two modifications; (1) the amplification program began with a 5 min activation step at 95°C, and five initial cycles with an incubation step of 30 s at 95°C, a 30 s annealing step at 42°C and a 1 min extension at 72°C, followed by 30 cycles with an incubation step at 95°C for 30 s, a 30 s annealing step at 50°C and a 1 min extension at 72°C. The products of this amplification were diluted 1:20 and used as template in successive reactions. In addition to the primers used by Litt and Irish (2003) the forward degenerate primer ATGGRDAGAGGWAGGGTWCAG, designed to bind the beginning of the MADS domain, was used in combination with all degenerate reverse primers designed to amplify the full coding sequence towards the 5′ end of the *FUL-like* genes. All PCR products were run on a 1% agarose gel and amplicons between 600 and 900 bp in size were cloned into pCR®2.1-TOPO® (Invitrogen). Clones were grown overnight, plasmid was extracted with the Qiagen miniprep Kit (Invitrogen) and sequenced at the DNA Yale Sequencing Center (CT).

In addition to degenerate PCR, we searched public databases, using BLAST (Altschul et al., 1990) and obtained 16 *FUL-like* genes from the transcriptomes available at the phytometasyn project website (http://www.phytometasyn.ca) and 29 *FUL-like* genes from GenBank (http://www.ncbi.nlm.nih.gov/genbank/). Sequences from 51 species and all families in Ranunculales (Eupteleaceae, Papaveraceae, Lardizabalaceae, Menispermaceae, Berberidaceae and Ranunculaceae) were included except Circaeasteraceae, from which material could not be obtained. Outgroups included representatives of the Magnoliaceae, Lauraceae, Saururaceae and Poaceae (Table S1).

### Phylogenetic analyses

Between 40 and 60 clones were sequenced per species. If variation was found among clones, the criteria to distinguish allelic variation at a single locus from different loci were the same used by Litt and Irish (2003). *FUL-like* sequences in the transcriptome databases were assembled into contigs and screened for polymorphisms using Sequencher DNA sequencing software (GeneCodes, Ann Arbor, MI): if different hits had less than 5% variation a consensus sequence was generated; if the difference among hits was larger, the two sequences were both kept in the analysis. Only sequences containing at least part of the MADS domain and the FUL-motif were included in the analysis. Sequences were compiled using Bioedit (http://www.mbio.ncsu.edu/bioedit/bioedit.html), and then aligned using the online version of MAFFT (http://mafft.cbrc.jp/alignment/server/) (Katoh et al., 2002), with a gap open penalty of 3.0, an offset value of 0.3, and all other default settings. The alignment was then refined by hand using Bioedit. The nucleotide alignment for 109 full-length sequences from 51 species was used for phylogenetic analyses. The amino acid alignment, also generated in Bioedit, was used to identify conserved motifs as well as single amino acids that were diagnostic of clades; these were optimized and visualized in MacClade4.08a® (Maddison and Maddison, 2005). The Magnoliid sequences (Ma.gr.AP1 and Pe.am.AP1) were used to root the trees, and all non-Ranunculid sequences were used as outgroup.

Maximum Likelihood (ML) phylogenetic analyses were performed in RaxML-HPC2 BlackBox (Stamatakis et al., 2008) on the CIPRES Science Gateway (Miller et al., 2009). The best performing evolutionary model was obtained by the Akaike information criterion (AIC; Akaike, 1974) using the program jModelTest v.0.1.1 (Posada and Crandall, 1998). Bootstrapping was performed according to the default criteria in RAxML where bootstrapping stopped after 200 replicates when the criteria were met.

### Relative rates of evolution

To test for evidences of changes in selection constraints in the Ranunculid *FUL-like* gene tree, we performed a series of likelihood ratio tests (LRTs) using the branch-specific model implemented by the CodeML program of PAML package v.4.6 (Yang, 2007). We compared the one ratio model that assumes a constant dN/dS ratio (= ω, per site ratio of nonsynonymous -dN- to synonymous -dS- substitution) along tree branches, against a two-ratio model that assumes a different ratio for a designated ranunculid *FUL-like* subclade (foreground) relative to the remaining sequences (background). For each of the LRTs, twice the difference of log likelihood between the models (2 Δ lnL) was compared to critical values from a χ 2 distribution, with degree of freedom equal to the differences in number of estimated parameters between models. The test was conducted for the entire dataset and also for each of the functional domains defined for MADS-box genes. These analyses on the M, IK, and C domains were performed in order to evaluate whether there was a difference in their rates of evolution in different taxa, given their key roles in DNA binding (M), protein dimerization (IK), and multimerization (C).

## Results

### *FUL-like* gene cloning in ranunculales

**Figure 2:**
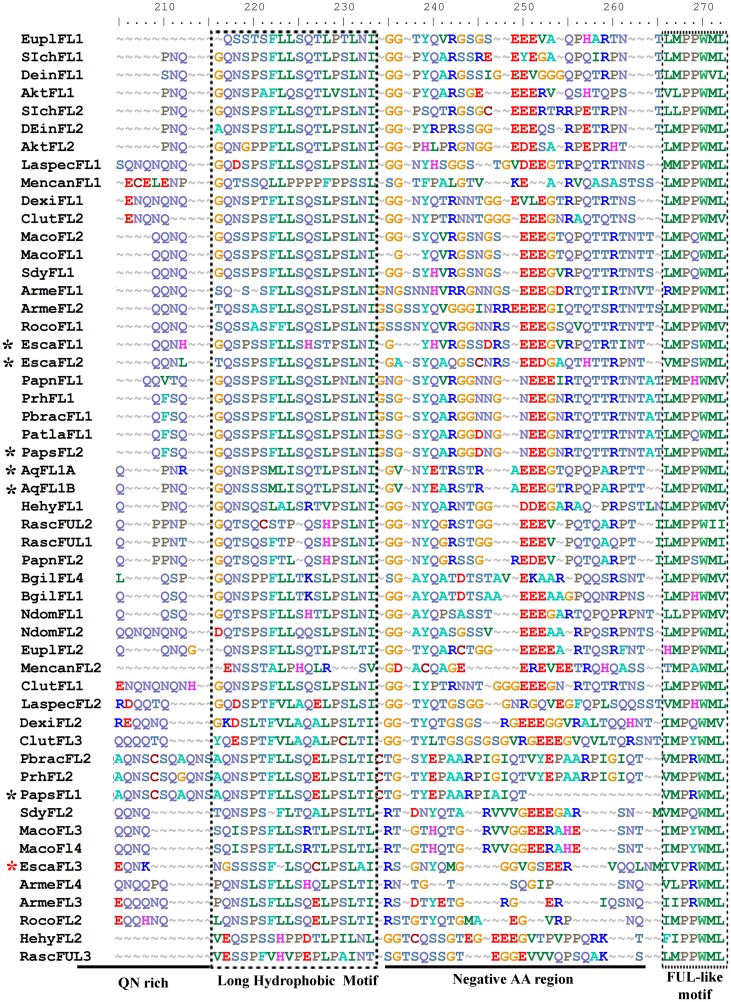
**Sequence alignment including the end of the K domain (K) and the complete C-terminal domain of ranunculid FUL-like proteins**. The alignment shows a region rich in glutamine (Q), asparagine (N) and serine (S), labeled as the QN rich zone, followed by the conserved hydrophobic motif newly identified (boxed), a region negatively charged and rich in glutamic acid (E), labeled the Negative AA region, and the FUL-like motif (boxed), typical of FUL-like and euFUL proteins. CmFL1 was excluded from the alignment because is the only sequence that has an additional insertion in the “hydrophobic motif” with 8 additional AA in between positions 229–236. Black asterisks show proteins that have been functionally characterized, red asterisk points to EscaFL3 that was not previously identified and has not been functionally characterized.

**Figure 3:**
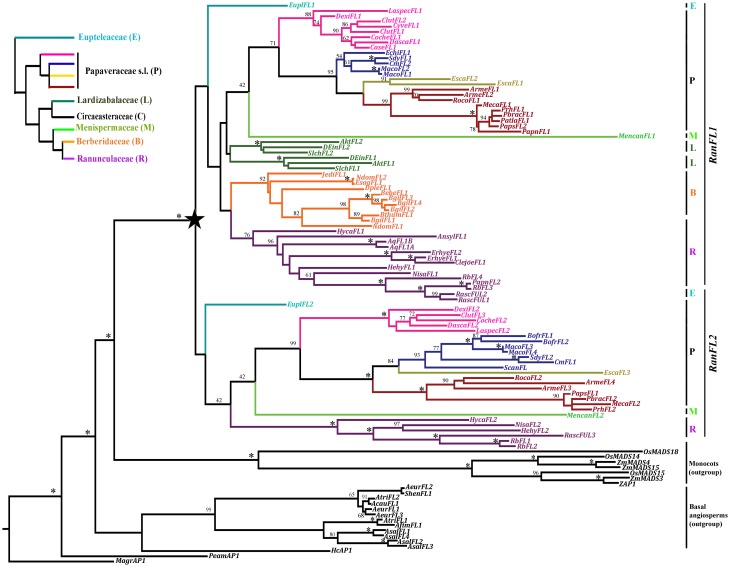
**Best Maximum Likelihood tree of *FUL-like* genes in Ranunculales. Bootstrap values (above 40%) are placed at nodes**. Asterisks indicate bootstrap values of 100%. The star indicates the duplication event that resulted in the Ran*FUL-like1* (*RanFL1*) and Ran*FUL-like2* (*RanFL2*) clades. Branch colors and vertical lines on the right denote different plant families as indicated on the organismal tree in the inset at the left (Wang et al., 2009). Papaveraceae s.l. is here shown with four different colors belonging to specific clades: bright pink shows the subfamily Fumarioideae; subfamily Papaveroideae is subdivided into the tribes Chelidonieae (blue), Eschscholtzieae (yellow) and Papavereae (red). Note that both the *RanFL1* and *RanFL2* clades have representative members from Eupteleaceae, Papaveraceae, Menispermaceae and Ranunculaceae, whereas, only *RanFL1* genes were amplified from Lardizabalaceae and Berberidaceae, suggesting that *RanFL2* genes from these families have been lost. In addition Lardizabalaceae *FL1* genes have undergone an independent duplication resulting in the Lardizabalaceae *FL1a* and *b* clades. B, Berberidaceae; E, Eupteleaceae; L, Lardizabalaceae; M, Menispermaceae; P, Papaveraceae; R, Ranunculaceae. Outgroup includes Basal angiosperms and Monocots in black.

In order to gain a better understanding of the basis of the functional diversity reported for *FUL-like* genes in the basal eudicot order Ranunculales, we looked at patterns of evolution among these genes. We isolated *FUL-like* copies from species representing the phylogenetic breadth of the Ranunculales, an order with nearly 202 genera and 4500 species (APG, 2009; Wang et al., 2009; Figures 2, 3), and reconstructed the evolutionary history of the gene lineage in this clade. Ranunculales includes the early-diverging families Eupteleaceae and Papaveraceae s.l., as well as the core Ranunculales Lardizabalaceae, Circaeasteraceae, Menispermaceae, Berberidaceae and Ranunculaceae. We generated a dataset consisting of 109 *FUL-like* gene sequences (Table S1) from Eupteleaceae, Papaveraceae s.l., Lardizabalaceae, Menispermaceae, Berberidaceae and Ranunculaceae, as well as the outgroup basal angiosperm and monocot families Magnoliaceae, Lauraceae, Saururaceae, Aristolochiaceae and the monocot family Poaceae. Sequences from Circeasteraceae were not included due to lack of availability of material.

Clones that were recovered with degenerate primers either span the entire coding sequence or are missing 10–20 amino acids (AA) from the start of the 60 AA MADS domain. The alignment includes 60 AA in the MADS domain, 35–40 in the I domain, 70–75 in the K domain, and 90 in the C-terminal domain. Among Ranunculales, paralogous gene sequence similarity ranges from 52 to 95%, and the variation in sequence similarity between outgroup and ingroup ranges from 50 to 75%. In the C-terminal portion, all protein sequences show the previously described FUL-like motif (Litt and Irish, 2003; Preston and Kellogg, 2006; Shan et al., 2007). Alignment of the predicted amino acid sequences of the entire dataset reveals a high degree of conservation in the M, I, and K regions until position 184. In most plant MADS proteins, the structurally conserved Keratin-like domain (K), forms three amphipathic helices (K1, K2, K3) that are important for strength and specificity of protein dimerization (Yang et al., 2003). Usually the three putative amphipathic α-helices of the K domain have heptad repeats (**a**bc**d**efg)_*n*_, in which **a** and **d** positions are occupied by hydrophobic amino-acids. The putative amphipathic α-helices of ranunculid FUL-like proteins, K1 (AA 97–110), K2 (AA 121–143) and K3 (AA 152–258), conform to this expected pattern. (Figure S1). Within K1, positions 99 (E), 102 (K), 104 (K), 106 (K), 108 (E), and 111 (Q), and within K2 positions 119 (G), 128 (K), 129 (E), 134 (E), 136 (Q), are conserved in all Ranunculales and outgroup FUL-like predicted protein sequences, with a few exceptions (Figure S1). The C-terminal domain, beginning after the hydrophobic amino acid located in position 184, is more variable, but three regions of high similarity can be identified: (1) a region rich in tandem repeats of polar uncharged amino acids (QNQ), particularly glutamine (Q), between positions 190–230 in the alignment; (2) a highly conserved, predominantly hydrophobic motif unique to ranunculids at positions 226–256, with the sequence QNS-P/LS/TFLLSQSE/LP-SLN/TI, and (3) a negatively charged region rich in glutamic acid (E) before the conserved FUL-motif LMPPWML (Figure 2).

### Gene duplication and loss of *FUL-like* genes in ranunculales

A total of 910 characters were included in the matrix, of which 645 (71%) were informative. Maximum likelihood analysis recovered a single duplication event early in the diversification of the Ranunculales resulting in two clades of *FUL-like* genes, here named *RanFL1* and *RanFL2* (Figure 3). Bootstrap support for the *RanFL1* and *RanFL2* clades is low (<50), however, within each clade, gene copies from the same family are grouped together with strong support, and the relationships among gene clades are mostly consistent with the phylogenetic relationships of the sampled taxa (Wang et al., 2009). An exception is the position of the Menispermaceae sequences as sister to the Papaveraceae s.l. sequences—although with long branches and low support—in both gene clades; phylogenetic analyses have shown Menispermaceae as the sister group to [Ranunculaceae + Berberidaceae] (Wang et al., 2009). Other inconsistent positioning is the placement of Lardizabalaceae as sister to [Papaveraceae + Menispermaceae], while it was sister to [Menispermaceae (Ranunculaceae + Berberidaceae)] in Wang et al. (2009).

Additional duplications and putative losses can also be detected. The *RanFL1* clade contains two paralogous Lardizabalaceae clades, *LarFL1a* and *LarFL1b*, but the *RanFL2* clade lacks sequences from this family. This suggests that *LarFL1* genes underwent an independent duplication, and that *LarFL2* members have been lost or are yet to be found. *RanFL2* sequences were also not recovered from Berberidaceae. Additional taxon-specific duplications were found in *Pseudofumaria lutea, E. californica* (Papaveraceae sl.), *Berberis gilgiana* and *Nandina domestica* (Berberidaceae), *A. coerulea, Eranthis hyemalis* and *Ranunculus sceleratus* (Ranunculaceae) within the *RanFL1* clade. Similarly, duplications were found in *Bocconia frutescens* (Papaveraceae) within the *RanFL2* clade. Finally, duplications in both clades (*RanFL1* and *RanFL2*) were evident in *Argemone mexicana, Macleaya cordata* (Papaveraceae), and *Ranunculus bulbosus* (Ranunculaceae). Since most of these species are thought to be polyploid (Index to Plant Chromosome Numbers; Missouri Botanical Garden, http://www.tropicos.org/Project/IPCN), additional duplicates are likely derived from whole genome duplications. If so, these transcription factors, that are thought to function as tetramers with other MADS box proteins at least in flower development (Smaczniak et al., 2012), are likely to maintain their functions and partners, given that during polyploidization events their partners also duplicate (Otto and Whitton, 2000; Blanc and Wolfe, 2004). Duplicates in *E. californica* are likely tandem-repeats or transcripts inserted by retro-transposition, as this is thought to be a diploid species with a chromosome number of 2n = 14 (Hidalgo et al., in prep). Similar local *FUL-like* gene duplications may have occurred in *E. hyemalis* and *R. bulbosus*, which are also thought to be diploids (2n = 16; Index to Plant Chromosome Numbers; Missouri Botanical Garden, http://www.tropicos.org/Project/IPCN).

Taxon-specific losses are harder to confirm, since is possible that some copies were not recovered through our cloning strategy. Nevertheless, our results suggest that *RanFL1* copies were lost in *Sanguinaria canadensis* and *B. frutescens* (Papaveraceae s.str.), and that *RanFL2* copies were lost in *Cysticapnos vesicaria, Capnoides sempervirens* and *Eomecon chionanta* (Papaveraceae s.l.) as well as in *Anemone sylvestris, E. hyemalis, Clematis* sp and *A. coerulea* (Ranunculaceae). The loss can only be confirmed in the case of *A. coerulea* as in this case the genome has been sequenced (Joint Genome Institute, 2010).

**Figure 4:**
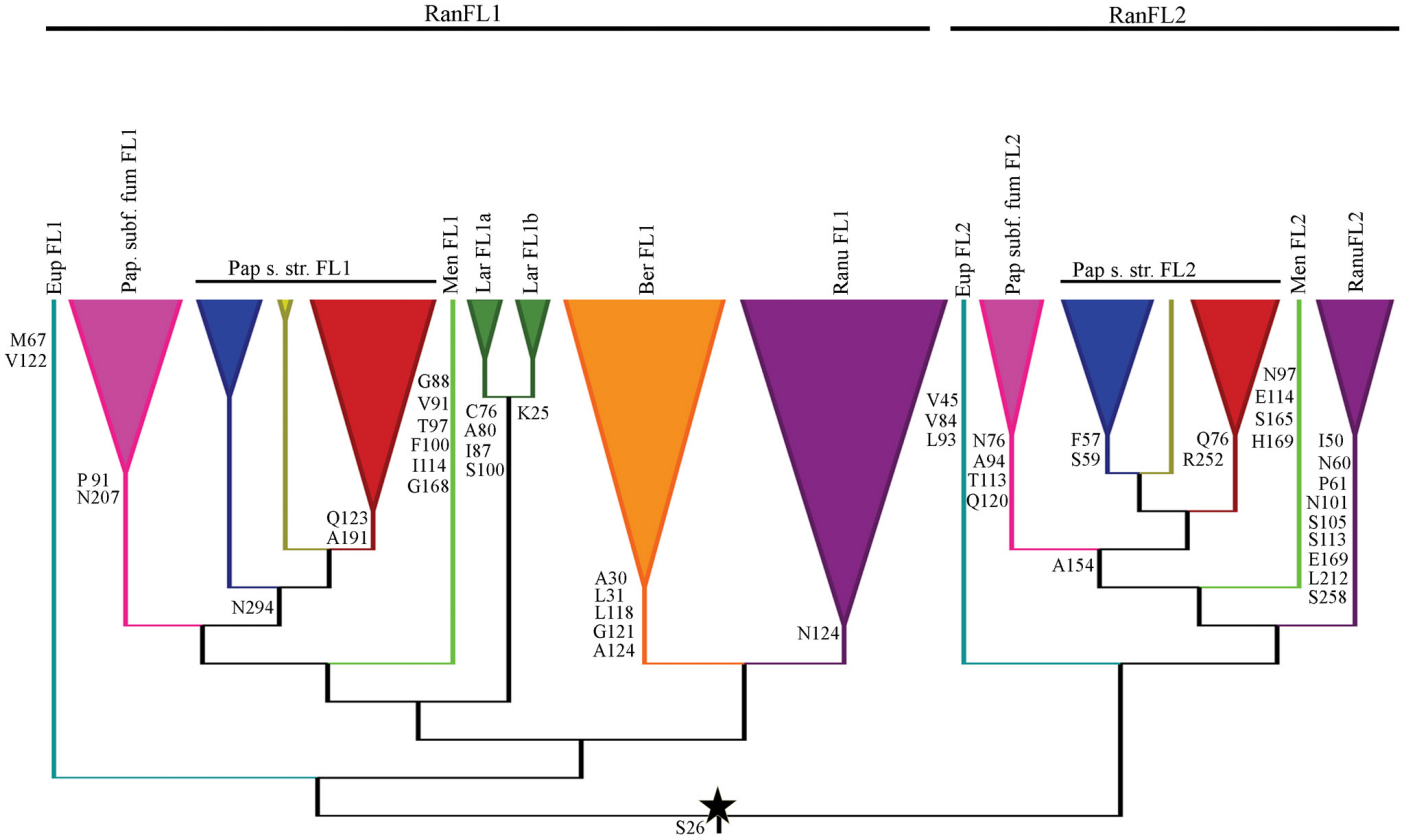
**Diagnostic amino acid characters of the Ranunculales FUL-like proteins, indicating the position in our alignments, mapped on a summary gene tree**. The star denotes the duplication event. Colors and names of the gene clades follow Figure 3 and are here abbreviated.

Finally we identified amino acid synapomorphies for subclades within the *RanFL1* and RanFL2 subclades, but no synapomorphies for those two clades themselves, consistent with the low support values in the deeper branches of the tree (Figures 3, 4). Nearly all the terminal subclades have at least one synapomorphy or as many as nine, however, the number of synapomorphies for each paralogous subclade differs greatly according to the family. For instance whereas Papaveraceae s. str. FL1 and FL2 have a single synapomorphy supporting each clade, Ranunculaceae FL1 and FL2 have one and nine synapomorphies respectively, suggesting that conserved aminoacids may have been fixed at different rates in the coding sequences of different paralogous clades.

### Shifts in selection constraints in the history of ranunculales *FUL-like* genes

**Figure 5:**
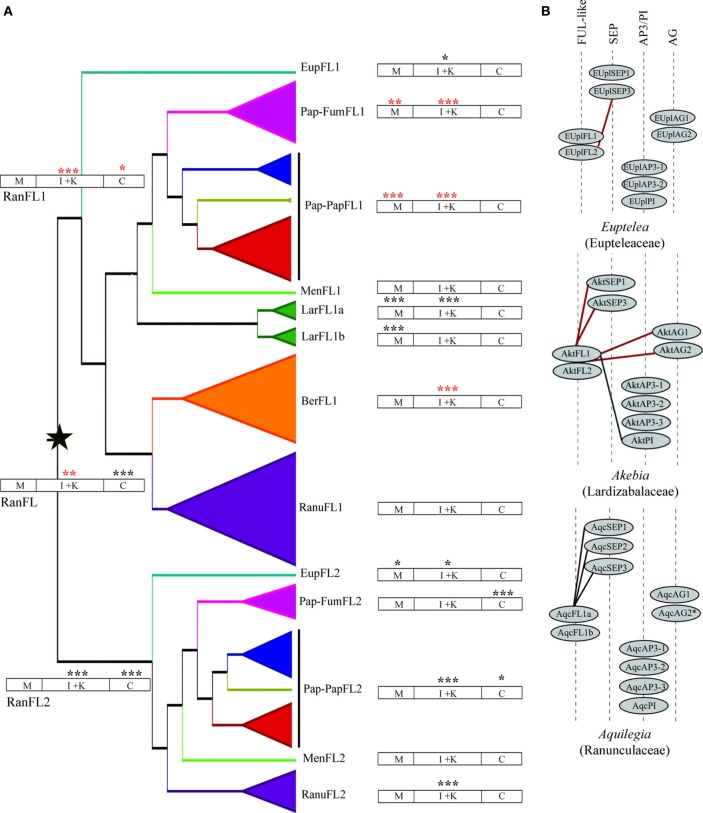
**(A)** Changes in selection constraint in the ranunculid *FUL-like* lineage inferred by the CodeML program of PAML. The star denotes the duplication event. The protein structure has been diagramed to show the MADS-box (M), the I and K (I + K), and the C-terminal (C) domains. The two-ratio model was tested on all ranunculid genes, the *RanFL1* and *RanFL2* clades, and all the subclades. Asterisks indicate which genes and which regions of the protein have a significantly better fit under the two-ratio model. The color of the asterisks indicates whether the proteins show an increase in the degree of purifying selection (red), or a relaxed degree of purifying selection (black). Significance: ^*^P < 0.05, ^**^P < 0.01, ^***^P < 0.001. **(B)** Summary of the reported protein interactions of ranunculid FUL-like genes with SEPALLATA (SEP), APETALA3/PISTILLATA (AP3/PI) and AGAMOUS (AG) floral organ identity proteins. Solid red lines indicate that both FUL-like copies were tested and had the same interactions. Solid black lines indicate that only that particular FUL-like copy was tested. Interactions are those reported in Liu et al. (2010) and Pabón-Mora et al. (2013).

Likelihood ratio tests, carried out to determine whether there were differences in selection acting on the ranunculid FUL-like sequences, show all tested ranunculid lineages to have ω < 1, indicating purifying selection (Table 1). This purifying pressure, however, exhibits significant variation (strengthening and release) across FUL-like subclades and in different protein domains (Figure 5A; Table 1). Indeed, while Ranunculales do not show a significant difference in the selective pressure acting on FUL proteins with respect to background taxa (basal angiosperms and grasses) at the level of the whole sequence, purifying pressure is significantly reinforced in the MADS domain and released in the IK region. In addition the analyses revealed that although both gene clades are under purifying selection, the degree of purifying selection is stronger in RanFL1 (ω_*f*_ = 0.18 vs. ω_*b*_ = 0.25) and significantly relaxed in RanFL2 (ω_*b*_ = 0.29 vs. ω_0_ = 0.19) (Figure 5A, ω_*f*_ vs. ω_*b*_ values and statistical significance are listed in Table 1).

**Table 1:**
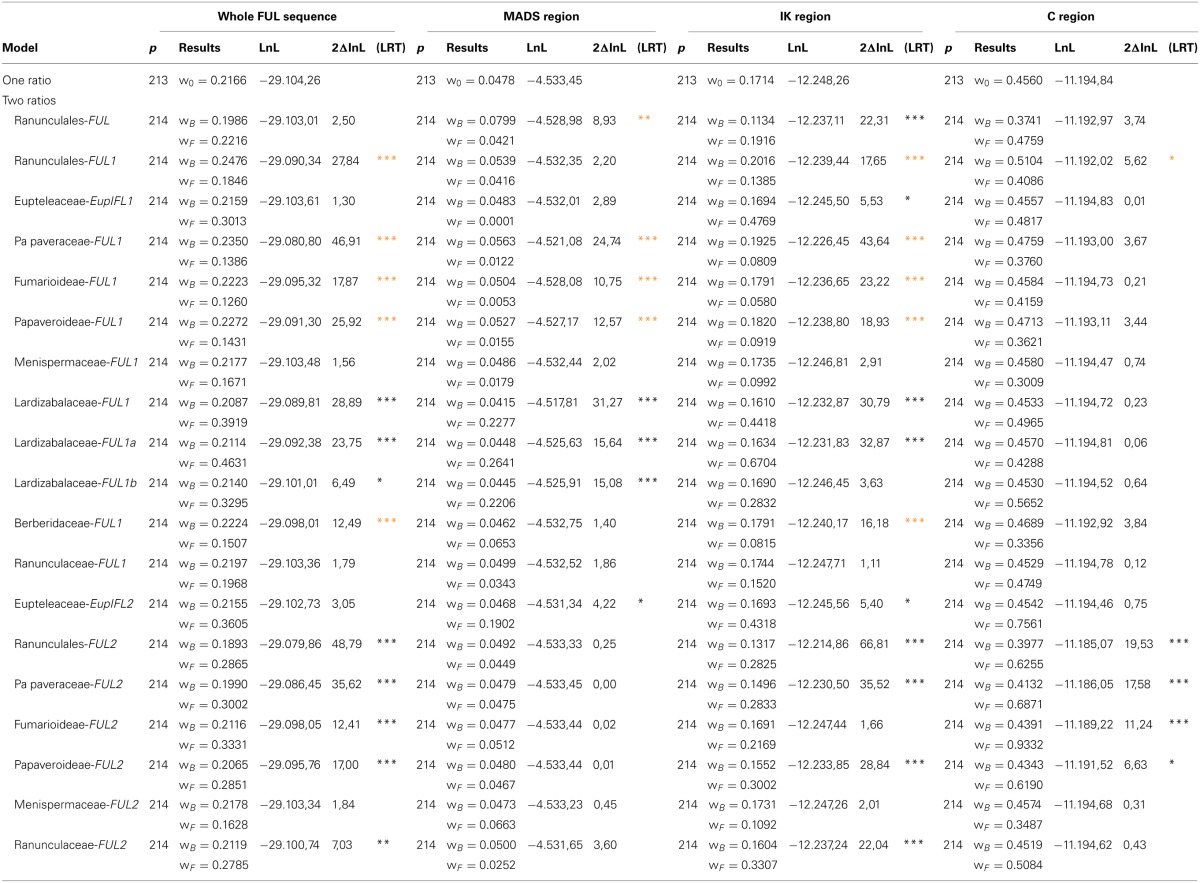
**Comparison of the one ratio model that assumes a constant dN/dS ratio (= ω, per site ratio of nonsynonymous -dN- to synonymous -dS- substitution) along tree branches, against a two-ratio model that assumes a different ratio for a designated ranunculid FUL-like subclade (foreground -ω_f_) relative to the remaining sequences (background -ω_b_)**.

For each of the LRTs, twice the difference of log likelihood between the models (2 Δ lnL) was compared to critical values from a χ 2 distribution, with degree of freedom equal to the differences in number of estimated parameters between models. The test was conducted for the entire dataset and also for each of the functional domains defined for MADS-box genes. These analyses were repeated on the M, IK, and C domains in order to evaluate whether there was a difference in their rates of evolution in different taxa, given their key roles in DNA binding (M), protein dimerization (IK) and multimerization (C). The color of the asterisks indicates whether the proteins show an increase in the degree of purifying selection (red), or a relaxed degree of purifying selection (black). Significance: ^*^P < 0.05, ^**^P < 0.01, ^***^P < 0.001.

An expanded analysis using the two-ratio test in each gene subclade showed a more complicated pattern of molecular evolution with each plant family showing unique selection constraints. Strengthening of purifying selection is detected in Papaveraceae s.l. FL1 and Berberidaceae FL1 (ω_*f*_ = 0.13 vs. ω_*b*_ = 0.23 and ω_*f*_ = 0.15 vs. ω_*b*_ = 0.22 respectively), whereas purifying selection is relaxed in Lardizabalaceae FL1a (ω_*f*_ = 0.46 vs. ω_*b*_ = 0.21) and FL1b (ω_*f*_ = 0.33 vs. ω_*b*_ = 0.21), Papaveraceae FL2 (ω_*f*_ = 0.30 vs. ω_*b*_ = 0.19) and Ranunculaceae FL2 (ω_*f*_ = 0.21 vs. ω_*b*_ = 0.27). In addition, these analyses also detected strong purifying selection in Menispermaceae FL1 and FL2 (ω_*f*_ = 0.16 vs. ω_*b*_ = 0.21 and ω_*f*_ = 0.16 vs. ω_*b*_ = 0.21 respectively) as well as relaxed purifying selection in Eupteleaceae FL1 and FL2 (ω_*f*_ = 0.30 vs. ω_*b*_ = 0.21 and ω_*f*_ = 0.36 vs. ω_*b*_ = 0.21 respectively), however, significant statistical support is lacking in these cases (Figure 5A; Table 1).

In order to test whether specific regions of the proteins were experiencing different selective pressures, we repeated the tests on the three distinct protein regions: the MADS (1–180 nt), the I + K (181–541 nt) and the C-terminal (542–910 nt) domains. The results showed that the MADS domain was under strong purifying selection in the Papaveraceae s.l. FL1 (ω_*f*_ = 0.01 vs. ω_*b*_ = 0.05) and under relaxed purifying selection in Lardizabalaceae FL1a and FL1b (ω_*f*_ = 0.26 vs. ω_*b*_ = 0.04 and ω_*f*_ = 0.22 vs. ω_*b*_ = 0.04 respectively) and in the Eupteleaceae FL2 (ω_*f*_ = 0.19 vs. ω_*b*_ = 0.04). Changes in selection were also evident in the I + K domains, showing strong purifying selection in Papaveraceae s.l. FL1 (ω_*f*_ = 0.08 vs. ω_*b*_ = 0.19) and Berberidaceae FL1 (ω_*f*_ = 0.08 vs. ω_*b*_ = 0.18) and a relaxed purifying selection in Eupteleaceae FL1 and FL2 (ω_*f*_ = 0.47 vs. ω_*b*_ = 0.16 and ω_*f*_ = 0.43 vs. ω_*b*_ = 0.17), Lardizabalaceae FL1a (ω_*f*_ = 0.67 vs. ω_*b*_ = 0.16), Papaveraceae FL2 (ω_*f*_ = 0.28 vs. ω_*b*_ = 0.15) and Ranunculaceae FL2 (ω_*f*_ = 0.33 vs. ω_*b*_ = 0.16). Significative changes in selection at the C terminus were only detected in Papaveraceae s.l. (ω_*f*_ = 0.62 vs. ω_*b*_ = 0.39) (Figure 5A; Table 1).

## Discussion

### *FUL-like* genes underwent duplication early in the diversification of the ranunculales

The ML analysis showed a single major duplication in the ranunculid FUL-like genes which gave rise to the *RanFL1* and *RanFL2* gene clades early in the diversification of the order Ranunculales. This duplication was not recovered in previous analyses of the AP1/FUL gene lineage (Litt and Irish, 2003; Shan et al., 2007). Although these analyses suggested major duplications occurred in the *FUL-like* genes in Ranunculales, it was not clear when they occurred. Our analyses on an expanded sample of Ranunculales clearly established that there was a single major event very early in the diversification of the order, however, is still unclear whether this duplication occurred before or after the divergence of Eupteleaceae. In fact, low support within each major clade and high similarity between EUplFL1 and EUplFL2 suggest that an alternative topology to Figure 3 tree would be possible, in which two independent duplications occurred, one within the Eupteleaceae and another after the divergence of the Eupteleaceae but before the diversification of all other Ranunculiids. This would be similar to the scenario found in the reconstruction of the evolutionary history of the *APETALA3* (*AP3*) genes in the Ranunculales, in which three duplications occurred: one in the Eupteleaceae and two in the remaining Ranunculales (Sharma et al., 2011). This indication that *FUL-like* and *AP3* genes underwent duplication events early in the diversification of most Ranunculales, before or right after the split of Eupteleaceae, suggests a possible ancestral genome-wide polyploidization event (Cui et al., 2006) in the Ranunculales, independent to the already well-established gamma-duplication in the core eudicots (Jiao et al., 2011; Vekemans et al., 2012).

In addition, whereas *RanFL1* genes are found in all the families of the order sampled so far, *RanFL2* genes were not found in Lardizabalaceae and Berberidaceae. This may be because in those two families our primers did not pick up *RanFL2* genes, or those genes are not expressed in leaf or floral tissue, or they were lost. None of these hypotheses can be rejected at this time, but after numerous amplification attempts with multiple degenerate primers specifically targeted to *RanFL2* genes, as well as extensive database searches, we favor the second and the third.

The clarification of orthology and paralogy of previously functionally characterized *FUL-like* genes sheds light on why these *FUL-like* genes might have both overlapping and unique functions (Figure 1). Our results show that *PapsFL2* and *EscaFL1* and *EscaFL2* are orthologs belonging to the *RanFL1* clade (Figure 3). On the other hand, *PapsFL1* is orthologous to *EscaFL3*, which was not discovered in previous studies in *E. californica* (Figure 3). These latter two genes belong to the *RanFL2* clade. These results suggest that the reason *escafl1-fl2* double mutants in *E. californica* did not show defects in cauline leaf development, flowering time and petal identity as did *papsfl1-fl2* mutants may be because *EscaFL3* is redundant for these functions (Pabón-Mora et al., 2012). Our results also confirm that the two *A. coerulea FUL-like* copies are the result of an independent duplication, as *AqcFL1A* and *AqcFL1B* are recent paralogs belonging to the *RanFL1* clade. *RanFL2* copies are not present in the *Aquilegia* genome. This gene loss may explain why results from functional analyses in poppies could not be extrapolated to *Aquilegia* (Pabón-Mora et al., 2012, 2013), and indeed probably suggests results from *Aquilegia* cannot even be applied to other members of Ranunculaceae. Gene loss in *Aquilegia* might have resulted in the rewiring of flower and fruit developmental networks such that *FUL-like* genes are excluded from roles in floral meristem identity, floral organ identity, or fruit development, and instead have been co-opted into leaf development. Nevertheless, it is also possible that *AqcFL1* residual transcript, or redundancy with other transcription factors masked the roles of *AqcFL1* genes in flower and fruit development in previous experiments (Pabón-Mora et al., 2013).

### Sequence changes in the C-terminal domain resulted in new motifs that might play roles in activation and protein multimerization capabilities

We have shown that ranunculid FUL-like proteins have, at the beginning of the C terminal domain, glutamine-rich segments carrying from 3 to 9 consecutive glutamines (Q) and 3–4 non-consecutive glutamines. Glutamine-rich motifs are also found in grass FUL-like proteins (Preston and Kellogg, 2006), and glutamine-rich domains in plants, carrying from 4 to 20 repeats, have been known to behave as transcription activation domains (Gerber et al., 1994; Schwechheimer et al., 1998; Xiao and Jeang, 1998; Wilkins and Lis, 1999; Immink et al., 2009); this suggests that FUL-like proteins may have transcription activation capability similar to euAP1 proteins (Cho et al., 1999). However, AqFL1A and AqFL1B (with 2 consecutive and 2 non-consecutive Q), as well as PapsFL1 and PapsFL2 (both with 4 consecutive Q) have not been shown to auto-activate in yeast systems (Pabón-Mora et al., 2012, 2013). Other ranunculid FL proteins, like those of *Eschscholzia*, have a larger number of glutamines but have not yet been tested for transcription activation capability. Glutamine repeats in eukaryotes have also been hypothesized to behave as “polar zippers” in protein-protein interactions (Perutz et al., 1994; Michleitsch and Weissman, 2000), thus these regions might mediate strength and specificity of FUL-like protein interactions.

This study identified two additional protein regions conserved in ranunculid FUL-like proteins including the sequence QNS-P/LS/TFLLSQSE/LP-SLN/TI, and a negatively charged region rich in glutamic acid (E) before the conserved FUL-motif LMPPWML (Figure 2). There are no functional studies specific for these regions, however, it has been shown that the N/SS at positions 227–228 are consistently found in AP1/FUL proteins and shared with SUPPRESSOR OF OVEREXPRESSION OF CONSTANS 1 (SOC1) and some SEPALLATA proteins, and that mutations in these amino acids influence interaction specificity and can result in changes in protein partners (Van Dijk et al., 2010).

### Release of purifying selection in the I+K protein domains might have influenced functional diversification

Variation in the rates of evolution of different FUL-like protein regions may also explain the functional differences among characterized proteins in different species. This is based on the premise that the rate of amino acid substitution is limited by functional or structural constraints on proteins (Liu et al., 2008). Previous studies have shown that differences in the rates and patterns of molecular evolution appear to be associated with divergence of developmental function between paralogous MADS-box loci (Lawton-Rauh et al., 1999). A common way to measure changes in protein sequence evolution is the dN/dS ratio, which calculates the ratio of non-synonymous to synonymous changes in protein sequences and provides an estimate of selective pressure. A dN/dS < 1 suggests that strong purifying selection has not allowed for fixation of most amino acid substitutions, dN/dS > 1 suggests that constraints are reduced and new amino acids have been able to become fixed due to positive selection, and dN/dS = 1 suggests neutral evolution, in which synonymous changes occur at the same rate as non-synonymous changes and fixation of new amino acids occurs at a neutral rate (Li, 1997; Hurst, 2002).

Our results show that strong purifying selection can be detected in the RanFL1 clade compared to more relaxed purifying selection in the RanFL2 proteins (*p* < 0.001). This would suggest that RanFL2 proteins are evolving at a faster rate, having been released from strong purifying selection after the duplication, and suggests a scenario of long-term maintenance of ancestral functions in one clade (RanFL1) and sub or neo-functionalization in the other clade (RanFL2), (Aagaard et al., 2006). When the same analyses are applied to the subclades within *RanFL1* and *RanFL2*, this pattern can also be seen for the duplicates in Papaveraceae s.l. and Ranunculaceae, but not in other families. For instance a contradictory pattern is found in Lardizabalaceae, in which both FL1a and FL1b proteins (paralogous clades within RanFL1) show relaxed purifying selection, suggesting that within this family, ancestral *FUL*-like gene functions may have been redistributed among the paralogs or lost, with the potential for new functions to appear in the evolutionary process (Force et al., 1999; Conant and Wagner, 2002).

Our analyses also showed that relaxation in purifying selection occurred preferentially in the I + K domains (in Eupteleaceae FL1, FL2, Lardizabalaceae FL1a, FL1b, Papaveraceae s str. FL2 and Ranunculaceae FL2), where dimerization functions have been localized, and less frequently in the MADS domain (in Lardizabalaceae FL1 a and FL1b), important for DNA binding, and the C terminus (in Papaveraceae s str. FL2), the function of which is not known. Most protein motifs maintained in MADS box duplicates and involved in dimerization occur at a hot-spot at the junction between the MADS and the I domain and is clear that non-synonymous changes in this region can dramatically change protein interactions (Van Dijk et al., 2010). For instance, three spots between the MADS and the I domain are maintained in most MADS box proteins and are thought to control DNA binding, these include Alanine A57, Asparagine N60 and Methionine M61 (Van Dijk et al., 2010). In FUL-like proteins the A57 is replaced by another hydrophobic amino-acid more often Tyrosine Y or Phenylalanine F, the M61 appears in position M63 and is conserved in all sequences, and finally the hydrophobic N60 is maintained in Ranunculaceae FL2, but changed in the rest of RanFL2 and RanFL1 proteins for Aspartic Acid D. The importance of the IK domains in protein-protein interactions has been long recognized. For instance, the end of the I domain and the entire K domain have been identified as the most important regions for the interactions between FUL-like and SEPALLATA proteins in rice (Moon et al., 1999). Likewise, residues in position 148–158 in APETALA1 seem to be crucial for recovery of floral meristem identity (Alvarez-Buylla et al., 2006) and a point mutation in Y148N is known to cause the loss of interaction between AP1 and SEPALLATA4, AGAMOUS-Like6 and AGAMOUS-Like15 (Van Dijk et al., 2010). Altogether the data suggests that changes in the IK regions might be key in explaining the different functions reported in ranunculid FUL-like proteins *via* changes in protein interactions. This is in agreement with observations in paralogous regulatory genes in which relaxed purifying selection is associated with the partitioning or even the acquisition of new interacting protein partners compared to the ancestral (pre-duplication) protein interactions (Dermitzakis and Clark, 2001; see also He and Zhang, 2006; Wagner and Zhang, 2011). A comparison of protein-protein interaction data gathered from ranunculid FUL-like proteins and the outgroup Poaceae proteins partially supports this hypothesis. Protein interactions in grasses show that *Oryza sativa* FUL-like proteins OsMADS14, OsMADS15 and OsMADS18 can only interact with a narrow set of floral organ identity proteins, the SEPALLATA proteins (Moon et al., 1999). Similarly, the *Euptelea* FUL-like proteins (EuplFL1 and EuplFL2) only interact with SEPALLATA proteins (Liu et al., 2010). The same interactions with floral organ identity proteins have been recorded for *Aquilegia* (AqFL1a) FUL-like proteins (Pabón-Mora et al., 2013), under strong purifying selection. In contrast, *Akebia* (Lardizabalaceae) FUL-like proteins, under relaxed purifying selection, appear to have been able to expand the repertoire of protein partners and can interact with SEPALLATA, PISTILLATA and AGAMOUS orthologs (Liu et al., 2010). Clearly more data are required to test the hypothesis that Ranunculales FUL-like protein interactions are maintained under strong purifying selection but diverge under relaxed selection, with resulting diversification of functional outcomes (Figure 5B).

The data presented here and in previous publications (Pabón-Mora et al., 2012, 2013) allow us to hypothesize that: (1) *FUL-like* genes across ranunculids perform overlapping and unique roles in a manner that cannot be predicted by their expression patterns. (2) Variation in function is possibly due to key amino acid changes in the I and K domains, important in dimerization, as well as unique protein motifs in the C-domain likely important for multimerization. In combination, these might have provided FUL-like homologs in the Ranunculales with different biochemical capabilities and protein interactions. (3) Understanding the evolution of gene pleiotropy in terms of protein regions that might be important for different functions in pre-duplication *FUL-like* genes across basal eudicots, provides clues on how *FUL-like* genes might have taken on different roles. Future directions include expression analyses and functional characterization of *FUL-like* genes in other Ranunculales, tests on the protein interactions between FUL-like proteins and other floral organ identity proteins in different ranunculid taxa, and functional characterization of the conserved motifs, particularly at the IK domains and the C-terminus.

### Conflict of interest statement

The authors declare that the research was conducted in the absence of any commercial or financial relationships that could be construed as a potential conflict of interest.

## Supplementary material

Figure S1 **K-domain sequence alignment of ranunculid FUL-like proteins**. Hydrophobic amino-acids in the a and d positions in the heptad repeats (abcdefg)_*n*_ are in bold. The predicted protein sequence at this domain contains three amphipathic α-helices: K1, K2, and K3. Within K1, positions 99 (E), 102 (K), 104 (K) are conserved in all ranunculid sequences and the outgroup, except for Mencan1 y Mencan2. Similarly, positions 106 (K), 108 (E) are also conserved, except in RocoFL2, ArmeFL4. Finally 111 (Q) is also conserved except in MacoFL3, MacoFL4. Within K2 positions 119 (G), 128 (K), 129 (E), 134 (E), 136 (Q) are conserved except in ArmeFL3. Conserved hydrophobic amino-acids outside of the predicted helices are highlighted and labeled with h.

Table S1 **Accession numbers of *FUL-like* sequences used in this study**.
