# Supplementary material for: Assessing duplication and loss of APETALA1/FRUITFULL homologs in Ranunculales
Source: Front Plant Sci. 2013 Sep 17;4:358. doi: 10.3389/fpls.2013.00358 (PMC3775002; doi:10.3389/fpls.2013.00358)
Supplement: Figure S1 — K-domain sequence alignment of ranunculid FUL-like proteins. Hydrophobic amino-acids in the a and d positions in the heptad repeats (abcdefg)n are in bold. The predicted protein sequence at this domain contains three amphipathic α-helices: K1, K2, and K3. Within K1, positions 99 (E), 102 (K), 104 (K) are conserved in all ranunculid sequences and the outgroup, except for Mencan1 y Mencan2. Similarly, positions 106 (K), 108 (E) are also conserved, except in RocoFL2, ArmeFL4. Finally 111 (Q) is also conserved except in MacoFL3, MacoFL4. Within K2 positions 119 (G), 128 (K), 129 (E), 134 (E), 136 (Q) are conserved except in ArmeFL3. Conserved hydrophobic amino-acids outside of the predicted helices are highlighted and labeled with h. [file 60373__Presentation_1.PDF]

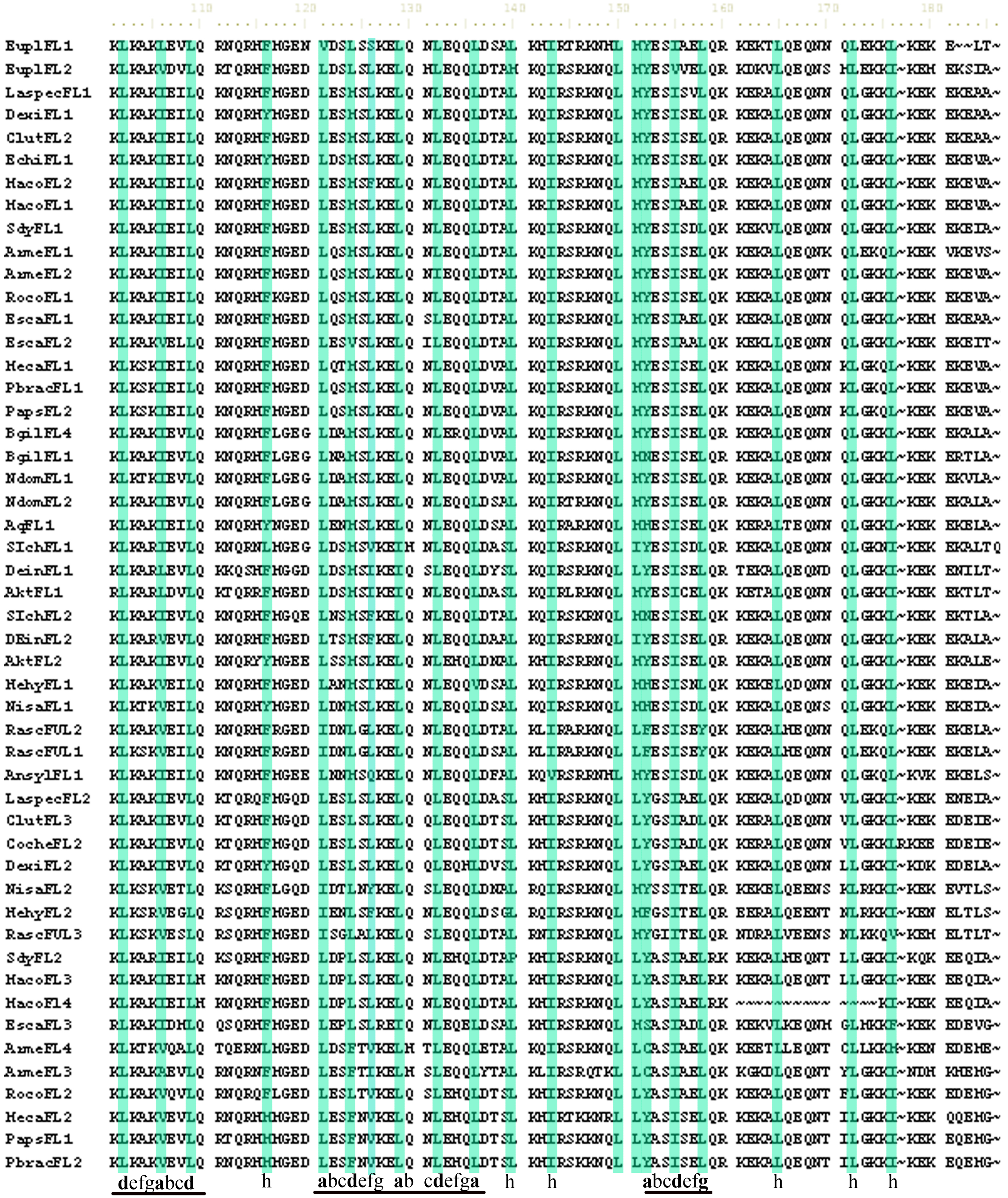
110 120 130 140 150 160 170 180
EuplFL1 KLKAKLEVLQ RNQRHFHGEN VDSLSSKELQ NLEQQLDSAL KHIKTRKNHL HYESIAELQR KEKTLQEQNN QLEKKL~KEK E~~LT~
EuplFL2 KLKAKVDVLQ KTQRHFHGED LDSLSLKELQ HLEQQLDTAH KQIRSRKNQL HYESVVELQR KDKVLQEQNS HLEKKI~KEH EKSIA~
LaspecFL1 KLKAKIEILQ KNQRHFHGED LESHSLKELQ NLEQQLDTAL KQIRSRKNQL HYESISVLQK KERALQEQNN QLGKKL~KEK EKEAA~
DexiFL1 KLKAKIEILQ KNQRHYHGED LESHSLKELQ NLEQQLDTAL KQIRSRKNQL HYESISELQR KEKALQEQNN QLGKKL~KEK EKEAA~
ClutFL2 KLKAKIEILQ KNQRHFHGED LESHSLKELQ NLEQQLDTAL KQIRSRKNQL HYESISELQR KEKALQEQNN QLGKKL~KEK EKEAA~
EchiFL1 KLKAKIEILQ KNQRHYHGED LDSHSLKELQ NLEQQLDTAL KQIRSRKNQL HYESISELQK KEKALQEQNN QLGKKL~KEK EKEVA~
HacoFL2 KLKAKIEILQ KNQRHFHGED LESHSFKELQ NLEQQLDTAL KQIRSRKNQL HYESIAELQR KEKALQEQNN QLGKKL~KEK EKEVA~
HacoFL1 KLKAKIEILQ KNQRHFHGED LESHSLKELQ NLEQQLDTAL KRIRSRKNQL HYESIAELQR KEKALQEQNN QLGKKL~KEK EKEVA~
SdyFL1 KLKAKIEILQ KNQRHFHGED LESHSLKELQ NLEQQLDTAL KQIRSRKNQL HYESISDLQK KEKVLQEQNN QLGKKL~KEK EKEIA~
AxmeFL1 KLKAKIEILQ KNQRHFHGED LQSHSLKELQ NLEQQLDTAL KQIRSRKNQL HYESISELQK KEKALQEQNK QLEKQL~KEK VKEVS~
AxmeFL2 KLKAKIEILQ KNQRHFHGED LQSHSLKELQ NIEQQLDTAL KQIRSRKNQL HYESISELQK KEKALQEQNT QLGKKL~KEK EKEVA~
RocoFL1 KLKAKIEILQ KNQRHFKGED LQSHSLKELQ NLEQQLDTAL KQIRSRKNQL HYESISELQK KEKALQEQNN QLGKKL~KEK EKEVA~
EscaFL1 KLKAKIEILQ RNQRHFHGED LQSHSLKELQ SLEQQLDTAL KQIRSRKNQL HYESIAELQK KEKALQEQNN QLGKKL~KEH EKEAA~
EscaFL2 KLKAKVELLQ RNQRHFHGED LESVSLKELQ ILEQQLDTAL KQIRSRKNQL HYESIAALQK KEKLLQEQNN QLGKKL~KEK EKEIT~
HecaFL1 KLKSKIEILQ KNQRHFHGED LQTHSLKELQ NLEQQLDVAL KQIRSRKNQL HYESISELQK KEKALQEQNN KLGKQL~KEK EKEVA~
PbracFL1 KLKSKIEILQ KNQRHFHGED LQSHSLKELQ NLEQQLDVAL KQIRSRKNQL HYESISELQK KEKALQEQNN KLGKQL~KEK EKEVA~
PapsFL2 KLKSKIEILQ KNQRHFHGED LQSHSLKELQ NLEQQLDVAL KQIRSRKNQL HYESISELQK KEKALQEQNN KLGKQL~KEK EKEVA~
BgilFL4 KLKAKIEVLQ KNQRHFLGEG LDAHSLKELQ NLERQLDVAL KQIRSRKNQL HYESISELQR KEKALQEQNN QLGKKL~KEK EKALA~
BgilFL1 KLKAKIEVLQ KNQRHFLGEG LNAHSLKELQ NLEQQLDVAL KQIRSRKNQL HNESISELQR KEKALQEQNN QLGKKL~KEK ERTLA~
NdomFL1 KLKTKIEVLQ KNQRHFLGEG LDAHSLKELQ NLEQQLDVAL KQIRSRKNQL HYESISELQR KEKALQEQNN QLGKKL~KEK EKVLA~
NdomFL2 KLKAKIEVLQ KNQRHFLGEG LDAHSLKELQ NLEQQLDSAL KQIKTRKNQL HYESISELQR KEKALQEQNN QLGKKL~KEK EKALA~
AqFL1 KLKAKIEILQ KNQRHYNGED LENHSLKELQ NLEQQLDSAL KQIRARKNQL HHESISELQK KERALTEQNN QLGKKL~KEK EKELA~
SIchFL1 KLKARIEVLQ KNQRNLHGEG LDSHSVKEIH NLEQQLDASL KQIRSRKNQL IYESISDLQR KEKALQEQNN QLGKNI~KEK EKALTQ
DeinFL1 KLKARLEVLQ KKQSHFHGGD LDSHSIKEIQ SLEQQLDYSL KQIRSRKNQL LYESISELQR TEKALQEQND QLGKKI~KEK ENILT~
AktFL1 RLKARLDVLQ KTQRRFHGED LDSHSIKEIQ NLEQQLDASL KQIRLRKNQL HYESICELQK KETALQEQNN QLGKKI~KEK EKTLT~
SIchFL2 KLKAKIEVLQ KNQRHFHGQE LNSHSFKELQ NLEQQLDTAL KQIRSKRNQL HNESISELQK KEKALQEQNN QLGKKL~KEK EKTLA~
DEinFL2 KLKARVEVLQ KNQRHFHGED LTSHSFKELQ NLEQQLDAAL KQIRSRRNQL IYESISELQR KEKALQEQNN QLGKKL~KEK EKALA~
AktFL2 KLKAKIEVLQ KNQRYYHGEE LSSHSLKELQ NLEHQLDNAL KHIRSRRNQL HYESISELQR KEKALQEQNN QLGKKL~KEK EKALE~
HehyFL1 KLKAKVEILQ KNQRHFHGED LANHSIKELQ NLEQQVDSAL KQIRSRKNQL HHESISNLQK KEKELQDQNN QLGKKL~KEK EKEIA~
NisaFL1 KLKTKVEILQ KNQRHYHGED LDNHSLKELQ NLEQQLDSAL KQIRSRKNQL HHESISDLQK KEKALQEQNS QLGKKL~KEK EKEIA~
RascFUL2 KLKAKVEILQ KNQRHFRGED IDNLGLKELQ NLEQQLDTAL KLIRARKNQL LFESISEYQK KEKALHEQNN QLEKQL~KEK EKELA~
RascFUL1 KLKSKVEILQ KNQRHFRGED IDNLGLKELQ NLEQQLDSAL KLIRARKNQL LFESISEYQK KEKALHEQNN QLEKQL~KEK EKELA~
AnsylFL1 KLKAKIEILQ KNQRHFHGEE LNNHSQKELQ NLEQQLDFAL KQVRSRRNHL HYESISDLQK KEKALQEQNN QLGKQL~KVK EKELS~
LaspecFL2 KLKAKIEVLQ KTQRQFHGQD LESLSLKELQ QLEQQLDASL KHIRSRKNQL LYGSIAELQK KEKALQDQNN VLGKKI~KEK ENEIA~
ClutFL3 KLKAKIEVLQ KTQRHFHGQD LESLSLKELQ QLEQQLDTSL KHIRSRKNQL LYGSIADLQK KERALQEQNN VLGKKI~KEK EDEIE~
CocheFL2 KLKAKIEVLQ KTQRHFHGQD LESLSLKELQ QLEQQLDTSL KHIRSRKNQL LYGSIADLQK KERALQEQNN VLGKKLRKEE EDEIE~
DexiFL2 KLKAKIEVLQ KTQRHYHGQD LESLSLKELQ QLEQHLDVSL KHIRSRKNQL LYGSIAELQK KEKALQEQNN LLGKKI~KDK EDELA~
NisaFL2 KLKSKVETLQ KSQRHFLGQD IDTLNYKELQ SLEQQLDNAL RQIRSRKNQL HYSSITELQR KEKELQEENS KLRKKI~KEK EVTLS~
HehyFL2 KLKSRVEGLQ RSQRHFHGED IENLSFKELQ NLEQQLDSGL RQIRSRKNQL HFGSITELQR EERALQEENT NLRKKI~KEN ELTLS~
RascFUL3 KLKSKVESLQ RSQRHFHGED ISGLALKELQ SLEQQLDTAL RNIRSRKNQL HYGIITELQR NDRALVEENS NLKKQV~KEH ELTLT~
SdyFL2 KLKARIEILQ KSQRHFHGED LDPLSLKELQ NLEHQLDTAP KHIRSRKNQL LYASIAELRK KEKALHEQNT LLGKKI~KQK EEQIA~
HacoFL3 KLKAKIEILH KNQRHFHGED LDPLSLKELQ NLEQQLDTAL KHIRSRKNQL LYASIAELRK KEKALQEQNT LLGKKI~KEK EEQIA~
HacoFl4 KLKAKIEILH KNQRHFHGED LDPLSLKELQ NLEQQLDTAL KHIRSRKNQL LYASIAELRK ~~~~~~~~~~ ~~~~KI~KEK EEQIA~
EscaFL3 RLKAKIDHLQ QSQRHFHGED LEPLSLREIQ NLEQELDSAL KHIRSRKNQL HSASIADLQR KEKVLKEQNH GLHKKF~KEK EDEVG~
AxmeFL4 KLKTKVQALQ TQERNLHGED LDSFTVKELH TLEQQLETAL KQIRSRKNQL LCASIAELQK KEETLLEQNT CLLKKH~KEN EDEHE~
AxmeFL3 KLKAKAEVLQ RNQRNFHGED LESFTIKELH SLEQQLYTAL KLIRSRQTKL LCASIAELQK KGKDLQEQNT YLGKKI~NDH KHEHG~
RocoFL2 KLKAKVQVLQ RNQRQFLGED LESLTVKELQ SLEHQLDTSL KHIRSRKNQL LYASIAELQK KEKALQEQNT FLGKKI~KEK EDEHG~
HecaFL2 KLKAKVEVLQ RNQRHHHGED LESFNVKELQ NLEHQLDTSL KHIKTKKNRL LYASISELQR KEKALQEQNT ILGKKI~KEK QQEHG~
PapsFL1 KLKAKVEVLQ KTQRHHHGED LESFNVKELQ NLEHQLDTSL KHIRSKKNQL LYASISELQR KEKALQEQNT ILGKKI~KEK EQEHG~
PbracFL2 KLKAKVEVLQ RNQRHHHGED LESFNVKELQ NLEHQLDTSL KHIRSKKNQL LYASISELQR KEKALQEQNT ILGKKI~KEK EQEHG~
defgabcd h abcdefg ab cdefga h h abcdefg h h h
