# Supplementary material for: Assessing duplication and loss of APETALA1/FRUITFULL homologs in Ranunculales
Source: Front Plant Sci. 2013 Sep 17;4:358. doi: 10.3389/fpls.2013.00358 (PMC3775002; doi:10.3389/fpls.2013.00358)
Supplement: Table S1 — Accession numbers of FUL-like sequences used in this study. [file 60373__Data_Sheet_1.DOCX]

**Suppl Table 1.** Accession numbers of *FUL-like* sequences used in this study.

In **bold** sequences identified in this work; *** sequences retrieved from BLAST; *~* sequences identified through http://www.phytometasyn.ca. ? Sequence that was previously identified as Papaver nudicaule FUL-like 2 but according to our analysis it belongs to a member of the Ranunculaceae, most likely Ranunculus. Source material for FUL-like gene amplification in this study comes from the The New York Botanical Garden and Lehman College.

| **Gene name** | **Species name** | **Family - Order** | **Accession number** |
| --- | --- | --- | --- |
| **OUTGROUP** | | | |
| *MagrAP1** | *Magnolia grandiflora* | Magnoliaceae - Magnoliales | AY821777 |
| *PeamAP1** | *Persea americana* | Lauraceae - Laurales? | DQ398019 |
| *HcAP1** | *Houttuynia cordata* | Saururaceae- Piperales | AB089153 |
| ***AcauFL1*** | *Asarum caudatum* | Aristolochiaceae - Piperales | KF500105 |
| ***AeurFL1*** | *Asarum europaeum* |  | KF500104 |
| ***AeurFL2*** |  |  | KF500103 |
| ***AeurFL3*** |  |  | KF500107 |
| ***ShenFL1*** | *Saruma henryi* |  | KF500102 |
| ***AtriFL1*** | *Aristolochia trilobata* |  | KF500112 |
| ***AtriFL2*** |  |  | KF500106 |
| ***AfimFL1*** | *Aristolochia fimbriata* |  | KF500113 |
| ***AsalFL1*** | *Aristolochia salvadorensis* |  | KF500108 |
| ***AsalFL2*** |  |  | KF500110 |
| ***AsalFL3*** |  |  | KF500111 |
| ***AsalFL4*** |  |  | KF500109 |
| **INGROUP** | | | |
| *EuplFL1** | *Euptelea pleiosperma* | Eupteleaceae - Ranunculales | DQ656558 |
| *EuplFL2** |  |  | DQ656559 |
| ***LaspecFL1*** | *Lamprocapnos spectabilis* | Papaveraceae subf. Fumarioideae - Ranunculales | KF500114 |
| ***LaspecFL2*** |  |  | KF500152 |
| ***DexiFL1*** | *Dicentra eximia* |  | KF500115 |
| ***DexiFL2*** |  |  | KF500155 |
| ***CocheFL1*** | *Corydalis cheilantifolia* |  | KF500119 |
| ***CocheFL2*** |  |  | KF500154 |
| ***ClutFL1*** | *Pseudofumaria lutea* |  | KF500117 |
| ***ClutFL2*** |  |  | KF500116 |
| ***ClutFL3*** |  |  | KF500153 |
| ***DascaFL1*** | *Dactylicapnos scandens* |  | KF500120 |
| ***DascaFL2*** |  |  | KF500151 |
| ***CaseFL1*** | *Capnoides sempervirens* |  | KF500118 |
| ***CyveFL1*** | *Cysticapnos vesicaria* |  | KF500121 |
| ***EchiFL1*** | *Eomecon chionantha* | Papaveraceae subf. Papaveroideae - Ranunculales | KF500122 |
| ***MacoFL1*** | *Macleaya cordata* |  | KF500124 |
| ***MacoFL2*** |  |  | KF500123 |
| ***MacoFL3*** |  |  | KF500158 |
| ***MacoFL4*** |  |  | KF500159 |
| ***BofrFL1*** | *Bocconia frutescens* |  | KF500160 |
| ***BofrFL2*** |  |  | KF500161 |
| *CmFL1** | *Chelidonium majus* |  | AY306144 |
| *CmFL2** |  |  | AY306145 |
| ***SdyFL1*** | *Stylophorum diphyllum* |  | KF500125 |
| ***SdyFL2*** |  |  | KF500156 |
| ***ScanFL*** | *Sanguinaria canadensis* |  | KF500157 |
| ***EscaFL1*** | *Eschscholzia californica* |  | HM592297 |
| ***EscaFL2*** |  |  | HM592298 |
| *EscaFL3 ~* |  |  | KF500168 |
| *ArmeFL1 ~* | *Argemone mexicana* |  | KF500126 |
| *ArmeFL2 ~* |  |  | KF500127 |
| *ArmeFL3 ~* |  |  | KF500163 |
| *ArmeFL4 ~* |  |  | KF500162 |
| ***RocoFL1*** | *Romneya coulteri* |  | KF500128 |
| ***RocoFL2*** |  |  | KF500164 |
| ***MecaFL1*** | *Meconopsis cambrica* |  | KF500129 |
| ***MecaFL2*** |  |  | KF500165 |
| ***PapsFL1*** | *Papaver somniferum* |  | AY306177 |
| ***PapsFL2*** |  |  | AY306178 |
| *PapnFL1** | *Papaver nudicaule* |  | AY306175 |
| *PapnFL2* | ? |  |  |
| ***PrhFL1*** | *Papaver rhoeas* |  | KF500130 |
| ***PrhFL2*** |  |  | KF500167 |
| *PbracFL1~* | *Papaver bracteatum* |  | KF500131 |
| *PbracFL2~* |  |  | KF500166 |
| ***PatlaFL1*** | *Papaver atlanticum* |  | KF500132 |
| *MencanFL1~* | *Menispermum canadense* | Menispermaceae- Ranunculales | KF500150 |
| *MencanFL2~* |  |  | KF500170 |
| *SIchFL1** | *Sinofranchetia chinensis* | Lardizabalaceae - Ranunculales | DQ656565 |
| *SIchFL2** |  |  | DQ656566 |
| *DEinFL1** | *Decaisnea insignis* |  | DQ656556 |
| *DEinFL2** |  |  | DQ656557 |
| *AktFL1** | *Akebia trifoliata* |  | AY627632 |
| *AktFL2** |  |  | GU357459 |
| *JediFL1~* | *Jeffersonia diphylla* | Berberidaceae - Ranunculales | KF500146 |
| *NdomFL1~* | *Nandina domestica* |  | KF500147 |
| *NdomFL2~* |  |  | KF500148 |
| *EsagFL1** | *Epimedium sagittatum* |  | JN590216 |
| *DpleFL1** | *Dysosma pleiantha* |  | JN593333 |
| *BebeFL1** | *Berberis bealei* |  | JN593334 |
| ***BthumFL1*** | *Berberis thumbergii* |  | KF500149 |
| ***BgilFL1*** | *Berberis gilgiana* |  | KF500145 |
| ***BgilFL2*** |  |  | KF500142 |
| ***BgilFL3*** |  |  | KF500143 |
| ***BgilFL4*** |  |  | KF500144 |
| *NisaFL1 ~* | *Nigella sativa* | Ranunculaceae - Ranunculales | KF500137 |
| *NisaFL2 ~* |  |  | KF500169 |
| *HycaFL1~* | *Hydrastis canadensis* |  | KF500136 |
| *HycaFL2~* |  |  | KF500172 |
| ***HehyFL1*** | *Helleborus hybrida* |  | KF500135 |
| ***HehyFL2*** |  |  | KF500171 |
| ***AqFL1A*** | *Aquilegia coerulea* |  | KF500133 |
| ***AqFL1B*** |  |  | KF500134 |
| ***AnsylFL1*** | *Anemone sylvestris* |  | KF500141 |
| ***ErhyeFL1*** | *Eranthis hyemalis* |  | KF500138 |
| ***ErhyeFL2*** |  |  | KF500140 |
| *RascFUL1** | *Ranunculus sceleratus* |  | AB473875 |
| *RascFUL2** |  |  | AB473876 |
| *RascFUL3** |  |  | AB473877 |
| *RbFL1** | *Ranunculus bulbosus* |  | AY306179 |
| *RbFL2** |  |  | AY306180 |
| *RbFL3** |  |  | AY306182 |
| *RbFL4** |  |  | AY306183 |
| ***ClejoeFL1*** | *Clematis sp. ‘cv joe’* |  | KF500139 |
